# Mitral valve repair for degenerative mitral regurgitation in patients with left ventricular systolic dysfunction: early and mid-term outcomes

**DOI:** 10.1186/s13019-020-01309-6

**Published:** 2020-10-01

**Authors:** Jun Li, Yun Zhao, Tianyu Zhou, Yongshi Wang, Kai Zhu, Junyu Zhai, Yongxin Sun, Hao Lai, Chunsheng Wang

**Affiliations:** 1 Department of Cardiovascular Surgery, Zhongshan Hospital, Fudan University, 180 Fenglin Road, Shanghai, 200032 China; 2 Shanghai Institute of Cardiovascular Diseases, Zhongshan Hospital, Fudan University, 180 Fenglin Road, Shanghai, 200032 China; 3 Shanghai Institute of Medical Imaging, Zhongshan Hospital, Fudan University, Shanghai, China; 4 Nuffield Department of Population Health, University of Oxford, Oxford, United Kingdom

**Keywords:** Degenerative mitral regurgitation, Mitral valve repair, Left ventricular systolic dysfunction

## Abstract

**Background:**

This study aims to evaluate the early and mid-term outcomes of mitral valve repair for degenerative mitral regurgitation (MR) in patients with left ventricular systolic dysfunction.

**Methods:**

From January 2005 to December 2016, the profiles of patients with degenerative MR who underwent mitral valve repair at our institution were analyzed. Left ventricular systolic dysfunction was defined as an ejection fraction < 60% or left ventricular end-systolic dimension > 40 mm. Finally, 322 patients with left ventricular systolic dysfunction were included in this study. The prognosis of left ventricular function during follow-up was evaluated and preoperative factors associated with deteriorated left ventricular systolic function during follow-up were analyzed.

**Results:**

The in-hospital mortality rate was 1.6%. The rate of eight-year overall survival, freedom from reoperation for mitral valve and freedom from recurrent MR were 96.9, 91.2 and 73.4%, respectively. Intraoperative residual mild MR (hazard ratio 4.82) and an isolated anterior leaflet lesion (hazard ratio 2.48) were independent predictive factors for recurrent MR. During follow-up, 212 patients underwent echocardiography examinations at our institution. Among them, 132 patients had improved left ventricular systolic function, and 80 patients had deteriorated left ventricular systolic. Freedom from recurrent MR was found in 75.9% of the improved left ventricular systolic function group and 56.2% of the deteriorated left ventricular systolic function group (*P* = 0.047). An age > 50 years (odds ratio 2.40), ejection fraction≤52% (odds ratio 2.79) and left ventricular end-systolic dimension≥45 mm (odds ratio 2.31) were independent risk factors for deteriorated left ventricular systolic function during follow-up.

**Conclusions:**

Mitral valve repair could be safely performed for degenerative MR in patients with left ventricular systolic dysfunction. Intraoperative residual mild MR and an isolated anterior leaflet lesion were independent predictive factors for recurrent MR. An age > 50 years, ejection fraction≤52% and left ventricular end-systolic dimension≥45 mm were independent risk factors for deteriorated left ventricular systolic function during follow-up.

## Background

Degenerative mitral valve (MV) disease is the most common cause of mitral regurgitation (MR) [1]. It is recommended by current guidelines that MV repair should be the primary choice to surgically treat degenerative MR [2–4]. Previous studies have demonstrated that early surgical intervention for degenerative MR significantly improves early and long-term survival [5, 6]. It has been recommended by the update of the ACC/AHA guidelines that surgery should be performed before left ventricular (LV) systolic dysfunction (class IIa) [4] in experienced centers.

Although early surgical intervention is beneficial for degenerative MR, patients presenting LV systolic dysfunction are not rare in clinical practice. Severe degenerative MR causes LV dilation which leads to more severe MR and increased LV volume overload. Long-term severe MR and LV volume overload is associated with high mortality and morbidity rates [7]; therefore, MV surgery is also necessary in patients with LV systolic dysfunction. However, the safety and effectiveness of MV repair in these patients has not yet been fully evaluated. Studies about the outcomes of patients with LV systolic dysfunction after MV repair are scarce, and the risk factors for mid- and long-term outcomes are unclear. In addition, it is not clear whether LV systolic dysfunction could be improved after MV repair.

This study aimed to analyze the safety and mid-term outcomes of mitral valve repair for degenerative MR in patients with LV systolic dysfunction. We further assessed the potential preoperative factors for deteriorated LV systolic function during follow-up.

## Materials and methods

### Patients

From January 2005 to December 2016, the profiles of all patients with degenerative MR who underwent MV repair at our institution were analyzed. Preoperative characteristics, operative details and perioperative results were acquired retrospectively. An isolated etiology of degenerative disease was confirmed by preoperative transthoracic echocardiography (TTE) and then verified by intraoperative transesophageal echocardiography (TEE) and surgical inspection. LV function was assessed by preoperative TTE. According to current guidelines [4], LV systolic dysfunction was defined as a LV ejection fraction (EF) < 60% or LV end-systolic dimensions> 40 mm. Except for patient who underwent tricuspid valvuloplasty, left atrial appendage closure and patent foramen ovale closure, patients who underwent other concomitant procedures including aortic valve repair/replacement, coronary artery bypass graft, atrial fibrillation ablation and congenital heart disease surgery etc. were excluded from the study. Finally, 322 patients with LV systolic dysfunction were included. The preoperative details are listed in Table 1.

**Table 1:** Preoperative profiles

Age	52.7 ± 12.7
Male	250 (77.6%)
NYHA function classification	
Class I	13 (4.0%)
Class II	109 (33.9%)
Class III	172 (53.4%)
Class IV	28 (8.7%)
AF	81 (25.2%)
Hypertension	137 (42.5%)
Diabetes Mellitus	32 (9.9%)
Coronary artery disease	13 (4.0%)
Chronic kidney disease	3 (0.9%)
Chronic obstructive pulmonary disease	8 (2.5%)
Stroke	7 (2.2%)
Preoperative echocardiography	
Ejection fraction	58.7 ± 7.3
LV end-systolic dimension	43.9 ± 5.9
LV end-diastolic dimension	64.9 ± 7.5
Left atrial dimension	54.9 ± 9.1
Pulmonary systolic pressure	52.5 ± 19.1

Follow-up information after discharge was obtained through the outpatient department, referring cardiologists and telephone contact. Profiles of overall survival, reoperation for MV and recurrent MR were obtained and analyzed. Recurrent MR was defined as moderate or severe MR detected by TTE during follow-up. The degree of MR was recorded as none or trivial, mild, moderate or severe.

We further analyzed LV systolic function during follow-up. According to follow-up TTE, we identified patients with improved LV systolic function (increased EF and decreased LV end-systolic dimension) and deteriorated LV systolic function (decreased EF or increased LV end-systolic dimension). To reduce bias, patients who underwent follow-up TTE examinations at other institutions were excluded. We compared differences in preoperative profiles and perioperative details between the improved LV systolic function group and deteriorated LV systolic function group, and then analyzed the potential predictive factors for deteriorated LV systolic function.

This study was reviewed and approved by the institutional review board of Zhongshan Hospital Fudan University and was conducted in accordance with the Declaration of Helsinki. The informed consent was waived.

### Operative technique

In our institution, we intended to perform MV repair in all patients with degenerative MR, and intraoperatively, TEE was routinely performed to evaluate MV lesions. Proper repair techniques were developed based on a combination of TEE results and surgical inspection. Quadrangular or triangular resection of the prolapse leaflet, artificial chordal reconstruction using expanded polytetrafluoroethylene sutures, and commissural closure were the primary leaflet repair techniques. An artificial annuloplasty band or ring was routinely implanted in 99.1% of patients.

Routine intraoperative TEE was performed subsequent to separation from cardiopulmonary bypass. Intraoperative reintervention was necessary for moderate or severe residual MR and systolic anterior motion which interfere with hemodynamics. Residual mild regurgitation was generally considered acceptable and did not require reintervention.

Minimally invasive surgery through right thoracotomy was performed in 43 patients. Patients with moderate/severe tricuspid regurgitation or dilation of the tricuspid annulus (> 40 mm) generally underwent additional tricuspid valvuloplasty. The operative techniques are presented in Table 2.

**Table 2:** Operative technique and perioperative outcomes

Minimal invasive surgery	43 (13.4%)
Tricuspid valvuloplasty	111 (34.5%)
Cardiopulmonary bypass time (min)	85.6 ± 27.2
Aorta cross clamp time (min)	49.9 ± 19.5
Primary repair techniques	
Leaflet resection	179 (55.6%)
Chordal reconstruction	66 (20.5%)
Commissural closure	59 (18.3%)
Ring	24 (7.5%)
Size	30.7 ± 2.1
Residual mild MR (TEE)	35 (10.9%)
Postoperative stroke	5 (1.6%)
Renal failure requiring renal replacement therapy	6 (1.9%)
Prolonged ventilation requiring tracheotomy	8 (2.5%)
Poor sternal wound healing	5 (1.6%)
Septicemia	3 (0.9%)
Low cardiac output syndrome	3 (0.9%)
Mediastinitis	1 (0.3%)
Length of stay (days)	7.5 ± 3.4
Intensive care unit (days)	1.8 ± 1.3
Thirty-day mortality	3 (0.9%)

### Statistics

Continuous variables are expressed as the mean ± standard deviation, and categorical variables are expressed as counts and percentages. The Kaplan-Meier method was used to compute survival curves. The Cox proportional hazards survival model was used to determine univariate and multivariate predictors of all cause death and recurrent MR. Potential predictors of all cause death and recurrent MR were tested in a univariate model and variables with *P* < 0.1 were included in the final Cox proportional survival analysis. Hazard ratios with the corresponding 95% confidence intervals (CI) were calculated.

Preoperative profiles were compared between the improved LV systolic function group and the deteriorated LV systolic function group. One-way analysis of variance and the χ2 test or Fisher’s exact test were used to compare continuous and categorical variables, respectively. For abnormally distributed variables, the Kruskal–Wallis test was used to identify differences. Receiver operating characteristic (ROC) curve analysis was used to identify the best cut-off values for preoperative EF, LV end-systolic dimension and end-diastolic dimension for predicting deteriorated LV function during follow-up. Logistic regression analysis was used to determine the univariate and multivariate risk factors for deteriorated LV function during follow-up. The discrimination of the final multivariate risk model was assessed with ROC analysis by computing the area under the curve (AUC). The calibration of the logistic models was assessed with the Hosmer-Lemeshow test. A *P* value < 0.05 was considered significant. All statistical analyses were conducted using SPSS software (version 22.0, IBM Corp., Armonk, NY).

## Results

The perioperative outcomes are shown in Table 2. The perioperative complications included stroke (5/322, 1.6%), acute kidney injury requiring renal replacement therapy (6/322, 1.9%), prolonged ventilation requiring tracheotomy (8/322, 2.5%), low cardiac output syndrome (3/322, 0.9%) and septicemia (3/322, 0.9%). The thirty-day mortality rate was 0.9% (3/322). The cause of death was low cardiac output syndrome in two patients, and multiple organ failure caused by acute kidney injury in one patient.

A total of 89% of the patients completed follow-up. The average follow-up time was 3.1 ± 2.6 years. Eight patients died during follow-up; the cause of death was cardio-related in three patients (heart failure in one and death after reoperation for MV in two), cerebrovascular accident in three patients, pneumonia in one patient and malignant tumor in one patient.

**Fig. 1:**
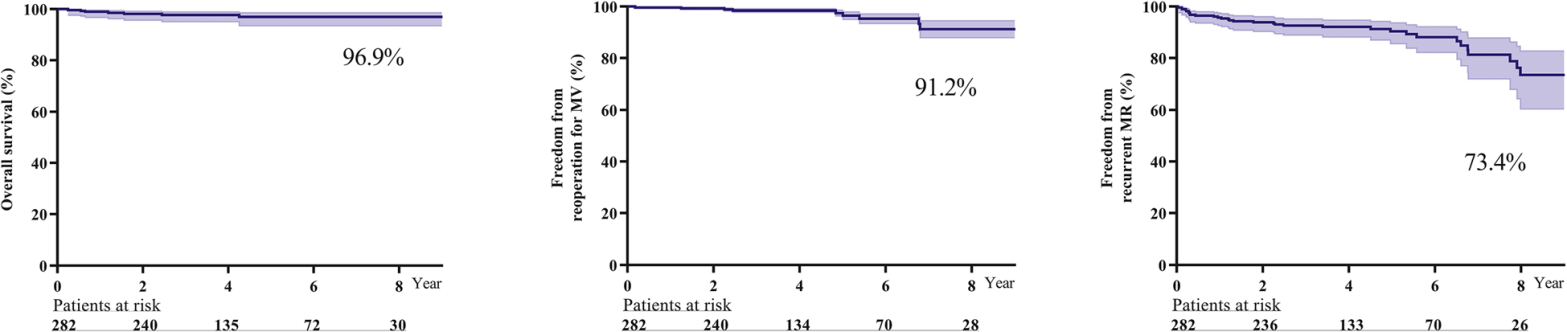
Kaplan-Meier curves. Eight-year over follow-up, overall survival, freedom from reoperation for mitral valve and freedom from recurrent mitral regurgitation were 95.2, 83.7 and 56.9%, respectively

Kaplan-Meier curves for the mid-term outcomes are shown in Fig. 1. The eight-year overall survival, freedom from reoperation for MV and freedom from recurrent MR were 96.9, 91.2 and 73.4%, respectively. In the Cox proportional hazard model, a pulmonary artery pressure > 55 mmHg (HR 6.07) and intraoperative residual mild MR (HR 8.00) were independent predictive factors for all-cause death during follow-up. Intraoperative residual mild MR (HR 4.82) and an isolated anterior leaflet lesion (HR 2.48) were independent predictive factors for recurrent MR during follow-up (shown in Table 3).

**Table 3:** Predictive factors for all cause death and recurrent MR using Cox proportional hazard model

Variables	Univariate model		Multivariable model	
	Hazard Ratio (95% CI)	*P* value	Hazard Ratio (95% CI)	*P* value
All cause death				
Age > 60 years	4.77 (1.14–20.01)	0.03	/	
PASP> 55 mmHg	4.79 (0.96–23.85)	0.06	6.07 (1.19–30.9)	0.03
Residual mild MR	6.45 (1.44–28.92)	0.02	8.00 (1.75–36.63)	0.007
Recurrent MR				
NYHA class III-IV	2.45 (1.06–5.68)	0.04	/	
Isolated anterior leaflet lesion	2.63 (1.30–5.32)	0.007	2.48 (1.22–5.04)	0.01
Residual mild MR	5.14 (2.40–11.03)	< 0.001	4.92 (2.26–10.68)	< 0.001

*NYHA* New York Heart Association, *PASP* Pulmonary artery systolic pressure, *MR* Mitral regurgitation

During follow-up, 212 patients underwent echocardiography examinations at our institution. Among them, 132 patients had improved LV systolic function, and 80 patients had deteriorated LV systolic function. The preoperative and operative details of these 212 patients are listed in Table 4. Older age, higher NYHA heart function classification, baseline atrial fibrillation, lower EF, and larger LV end-systolic dimension and end-diastolic dimension were associated with deteriorated LV systolic function during follow-up.

**Table 4:** Differences between improved and deteriorated LV function group

Variable	Improved LV function (*n* = 132)	Deteriorated LV function (*n* = 80)	P value
Age	50.5 ± 12.8	54.9 ± 11.7	0.01
Male	100 (75.8%)	66 (82.5%)	0.25
NYHA function classification			0.009
Class I	9 (6.8%)	2 (2.5%)	
Class II	53 (40.2%)	23 (28.8%)	
Class III	63 (47.7%)	46 (57.5%)	
Class IV	7 (5.3%)	9 (7.5%)	
AF	26 (19.7%)	27 (33.8%)	0.02
Hypertension	55 (41.7%)	41 (51.3%)	0.17
Diabetes Mellitus	13 (9.8%)	9 (11.3%)	0.75
Coronary artery disease	8 (6.1%)	4 (5.0%)	0.97
Chronic kidney disease	2 (1.5%)	1 (1.3%)	1
COPD	4 (3.0%)	3 (3.8%)	1
Preoperative stroke	3 (2.3%)	1 (1.3%)	0.99
Ejection fraction	60.0 ± 5.7	57.4 ± 7.9	0.01
LV end-systolic dimension	42.5 ± 4.6	45.5 ± 6.5	< 0.001
LV end-diastolic dimension	63.6 ± 6.7	66.0 ± 7.8	0.02
Left atrial dimension	54.1 ± 8.5	56.4 ± 9.6	0.08
Pulmonary systolic pressure	53.0 ± 19.6	49.6 ± 16.6	0.21
Tricuspid valvuloplasty	42 (31.8%)	30 (37.5%)	0.40
Leaflet resection	80 (60.6%)	43 (53.8%)	0.33
Chordal reconstruction	28 (21.2%)	13 (16.3%)	0.38
Commissural closure	29 (22.0%)	18 (22.5%)	0.93
Ring	8 (6.1%)	6 (7.5%)	0.69
Size	30.8 ± 2.0	30.8 ± 2.2	0.77
Cardiopulmonary bypass time (min)	85.8 ± 31.1	92.3 ± 26.8	0.16
Aortic cross clamp time (min)	50.6 ± 21.4	52.2 ± 19.1	0.60

*LV* Left ventricular, *NYHA* New York Heart Association, *AF* Atrial fibrillation, *COPD* Chronic obstructive pulmonary disease

In the ROC analysis, the cut-off values of 52.5% for preoperative EF, 44.5 mm for preoperative LV end-systolic dimension and 65.5 mm for preoperative LV end-diastolic dimension were the most useful for predicting deteriorated LV function during follow-up. The AUCs were 0.578 (95% CI 0.496–0.659), 0.643 (95% CI 0.563–0.724) and 0.593 (95% CI 0.512–0.674), respectively. In the multivariate logistic regression model, an age > 50 years (OR 2.40), EF ≤ 52% (OR 2.79) and LV end-systolic dimension≥45 mm (OR 2.31) were identified as independent risk factors for deteriorated LV function during follow-up (shown in Table 5). In the ROC analysis, the AUC was 0.682 (95% CI 0.605–0.758) for the multivariate logistic regression model. The *P* value of the model was 0.863 in the Hosmer and Lemeshow test.

**Table 5:** Risk factors for deteriorated LV function during follow-up using logistic regression model

	Odds Ratio (95% CI)	P value	Odds Ratio (95% CI)	P value
Variables	Univariate		Multivariate	
Age ≥ 50 years	2.72 (1.50–4.95)	0.001	2.40 (1.28–4.52)	0.007
Atrial fibrillation	2.02 (1.08–3.79)	0.03	/	
NYHA class III-IV	2.08 (1.16–3.72)	0.01	/	
EF ≤ 52%	2.79 (1.06–7.33)	0.04	2.79 (1.06–7.33)	0.04
LV end-systolic dimension≥45 mm	2.95 (1.63–5.34)	< 0.001	2.31 (1.20–4.46)	0.01
LV end-diastolic dimension≥66 mm	1.79 (1.02–3.16)	0.04	/	

*NYHA* New York Heart Association, *EF* Ejection fraction, *LV* Left ventricular

**Fig. 2:**
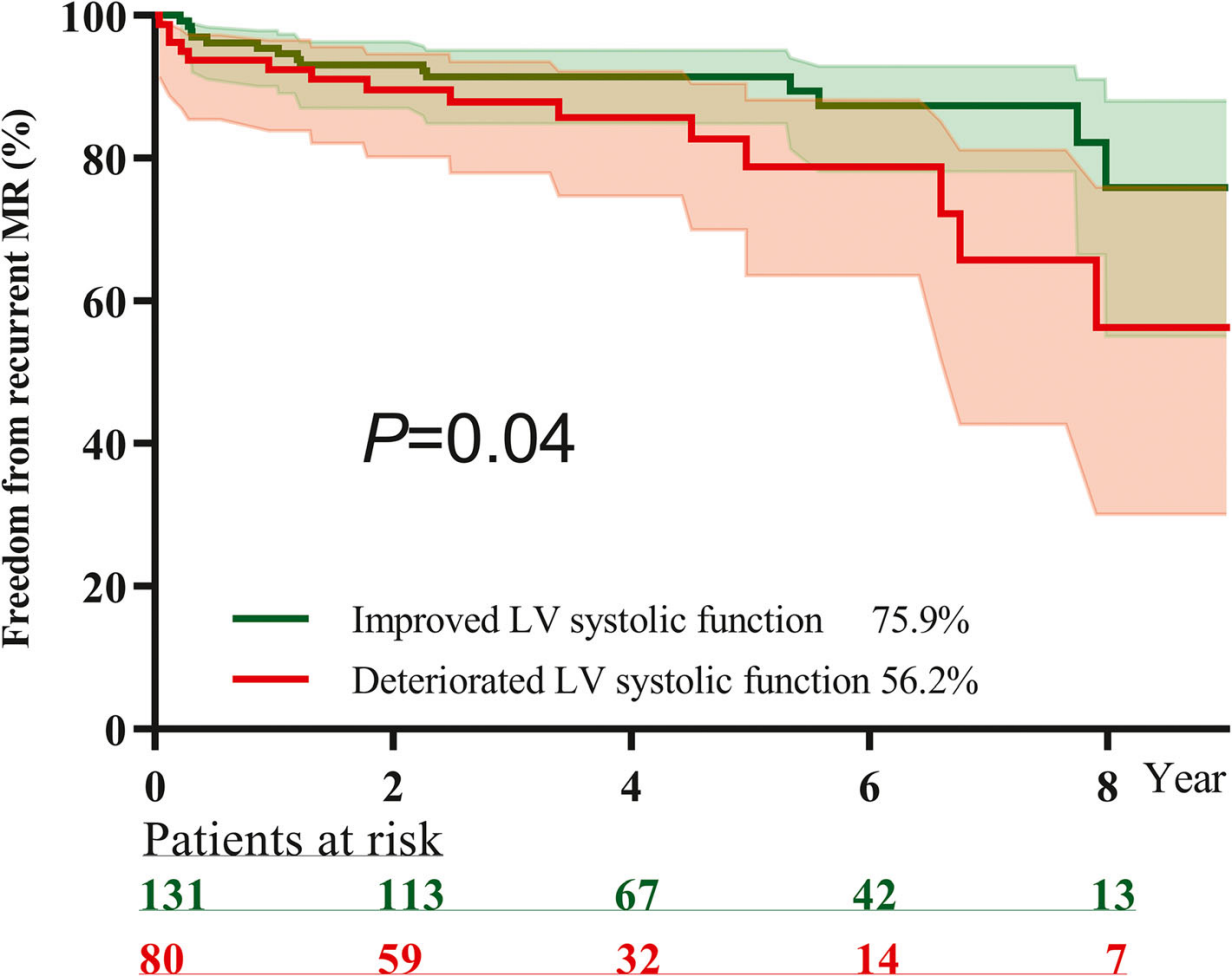
Comparison of freedom from recurrent MR between improved LV systolic function group (green line) and deteriorated LV systolic function group (red line) during follow-up. At eight years after operation, freedom from recurrent mitral regurgitation was 75.9% in improved LV systolic function group and 56.2% in deteriorated LV systolic function group (*P* = 0.04)

Freedom of recurrent MR was found in 75.9% of the improved LV systolic function group and 56.2% of the deteriorated LV systolic function group (*P* = 0.047). The Kaplan-Meier curve is shown in Fig. 2.

## Discussion

A previous study demonstrated that heart failure symptoms and a lower EF were associated with higher late mortality [1]. Therefore, in patients with LV systolic dysfunction, including a decreased ejection fraction and increased LV dimension, the effects of MV repair might be compromised. In this study, our results showed that in patients with LV systolic dysfunction, the incidence of perioperative complications was low, and thirty-day mortality was comparable to that in previous studies and our early study [8]. Previous studies have shown that the perioperative mortality rate of MV repair in degenerative MR varies from 0.2 to 1.2% [1, 6, 9, 10]. MV repair was associated with lower mortality than replacement in patients with or without LV dysfunction [11, 12]. These results demonstrated that MV repair could be safely performed in patients with LV systolic dysfunction, and should be preferred over replacement.

Regarding mid- and long-term outcomes, the 8-year overall survival rate was 96.9% in this study. Tirone David and colleagues showed in their study that the 10-year survival rate for degenerative MR after MV repair was 85.6% [1]. In in a study conducted by Coutinho et al., the 10-year survival rate for patients with preserved LV function was 89.7% [9]. In another study, Enriquez-Sarano demonstrated that in patients with heart failure symptoms, an EF < 60% and LV end-systolic dimension> 40 mm, the 10-year survival rate was only 64% [10]. Our results suggested that survival after MV repair in isolated degenerative MR patients with LV systolic dysfunction might not be severely compromised. In a previous study, Noack and colleagues evaluated isolated mitral valve repair in patients with reduced left ventricular ejection fraction. They found that the 5-year survival rate were 81.2, 75.2 and 58% in patients with EFs of 40–49%, 30–39% and < 30%, respectively. Considering that these patients experienced severe HF with a poor prognosis and quality of life, this result was acceptable [13]. Current opinions [6, 14] and guidelines for valve diseases recommend that early surgical intervention is reasonable for severe degenerative MR in asymptomatic patients with preserved LV systolic function, and that these methods could improve outcomes [2–4]. However, patients with LV systolic dysfunction are not uncommon in real world practice. For these patients, “salvage” surgical intervention is still safe, helpful and necessary to prevent further myocardial damage and adverse events.

In this study, the incidence of recurrent MR for patients with LV systolic dysfunction was relatively higher than that in other studies. An early study [15] suggested that the incidence of recurrent MR after MV repair in degenerative disease was high (28.9% at 7 years). However, the latest studies [1, 16] suggested that the durability of MV repair for degenerative MR was excellent, at approximately 10% at 10 years. Generally, complex valve lesions (anterior lesion, Barlow disease, etc.) and inappropriate repair techniques were considered to be associated with increased risk of recurrent MR [9, 16, 17]. We also found in our study that anterior leaflet prolapse and intraoperative residual mild MR were independent predictive factors for recurrent MR; however, in previous studies, patients with preserved LV systolic function constituted most of the patient cohorts. In our previous study [8], we found that recurrent MR was only 3% at 8-year during follow up for those who received early surgical intervention before the onset of guideline-based indications. Therefore, preoperative LV systolic dysfunction might be associated with recurrent MR. A dilated left ventricle with LV systolic dysfunction could lead to annular enlargement and apically displaced leaflets, which cause secondary MR. Therefore, persistent LV systolic dysfunction might be a potential cause of recurrent MR. On the other hand, residual mild MR was not only a predictive factor for recurrent MR but was also associated with a higher risk of all-cause mortality. Interestingly, the incidence of recurrent MR was significantly higher in the deteriorated LV systolic function group than in the improved LV systolic function group. Although it was difficult to determine which factor initiated the process, progressive MR and LV systolic function could cause a perpetual cycle and lead to poor prognosis. This highlighted the necessity to avoid intraoperative residual MR, especially in patients who already developed LV dysfunction.

Our study found that deteriorated LV systolic function occurred in approximately 38% of patients. Previous studies have demonstrated that LV systolic dysfunction after MV repair is not uncommon, even in patients with preserved preoperative LV systolic function [18–20]. More importantly, early postoperative LV systolic dysfunction might be persistent and associated with poor long-term outcomes [18, 20]. In patients with an abnormal EF and enlarged LV dimensions, abnormal myocardial contractile function existed for a significant period. Although abnormal hemodynamics could be corrected by repair operations, LV systolic dysfunction might not be improved due to its prolonged disease stage. Therefore, in patients with preoperative LV systolic dysfunction, it is more important to identify the potential predictors for deteriorated LV systolic function. In this study, an age > 50 years, EF ≤ 52% and LV end-systolic dimension≥45 mm were identified as independent risk factors by ROC analysis and a logistic regression model for deteriorated LV systolic function during follow-up. Therefore, MV repair should not be delayed in these patients in order to reverse LV systolic dysfunction. Nevertheless, the incidence of recurrent MR and deteriorated LV function during follow-up was relatively high for patients with preoperative LV systolic function, therefore close follow-up was necessary in these patients.

Our study found that more severe heart failure symptoms and preoperative atrial fibrillation were associated with deteriorated LV systolic function, although they were not independent risk factors in multivariate analysis. Several previous studies [19, 21] have analyzed the determinants of LV systolic dysfunction after MV repair and also found that preoperative atrial fibrillation and pulmonary hypertension were predictive factors for LV systolic dysfunction as well. Atrial fibrillation and pulmonary hypertension are both signs of the severity of MR and predict a poor prognosis [22]. Therefore, closer follow-up was necessary for patients who presented with a combination of LV systolic dysfunction, severe heart failure function and atrial fibrillation.

In a previous study [23], Imasaka and colleagues suggested that chordal replacement might be associated with better postoperative LV function than leaflet resection in the repair of posterior leaflet prolapse. However, in other studies [19–21], the factors of lesion segment and repair technique were not observed to be related to postoperative LV systolic dysfunction. Our results also suggested that the repair techniques were similar between the improved and deteriorated LV systolic function groups. Nevertheless, due to the potential relationship between recurrent MR and deteriorated LV systolic function, intraoperative residual mild MR should be avoided and managed carefully. In addition, close follow-up and postoperative echocardiography examinations are necessary for patients with LV systolic dysfunction. In the setting of persistent or deteriorated LV systolic function, the administration of standard anti-heart failure treatment is reasonable.

### Limitations

The present study was observational and retrospective, with the associated biases. Our single center’s experience might not apply to other institutions. Additionally, as a large tertiary hospital, we treat patients that come from all areas of the country. Therefore, the follow-up rate was negatively affected by the poor compliance of some patients from remote areas. Hence, approximately 25% of the follow-up echocardiographic examinations were performed at other institutions. Because we could not confirm every echocardiographic report generated at other institutions, some recurrent cases might have been missed detection. To maintain consistency between preoperative and follow-up echocardiography studies, the analysis of follow-up echocardiography profiles was only conducted for patients who underwent the examinations at our institution. The results might be affected by the sample selection. In addition, the mean follow-up time was relatively short in the present study. Further follow-up is needed to evaluate repair durability and LV systolic function in these patients.

## Conclusion

For patients witn degenerative MR with LV systolic dysfunction, MV repair could be safely performed with excellent mid-term survival outcomes. Intraoperative residual mild MR and an isolated anterior leaflet lesion were independent predictive factors for recurrent MR. An age > 50 years, EF ≤ 52% and LV end-systolic dimension≥45 mm were independent risk factors for deteriorated LV systolic function during follow-up.

## Data Availability

The datasets used and analyzed during the current study are available from the corresponding author on reasonable request.

## Abbreviations

MV: Mitral valve
MR: Mitral regurgitation
LV: Left ventricular
EF: Ejection fraction
NYHA: New York Heart Association
TTE: Transthoracic echocardiography
TEE: Transesophageal echocardiography
CI: Confidential interval
ROC: Receiver operating characteristic curve
AUC: Area under curve
HR: Hazard ratio
